# Minimal Residual Disease-Adapted Therapy in Multiple Myeloma: Current Evidence and Opinions

**DOI:** 10.1007/s11912-024-01537-2

**Published:** 2024-04-27

**Authors:** Mina Meseha, James Hoffman, Dickran Kazandjian, Ola Landgren, Benjamin Diamond

**Affiliations:** Myeloma Institute, Sylvester Comprehensive Cancer Center, University of Miami, 1120 NW 14th Street, Clinical Research Building, Miami, FL 33136 USA

**Keywords:** Multiple myeloma, Minimal residual disease, Flow cytometry, Next generation sequencing

## Abstract

**Purpose of Review:**

Multiple myeloma (MM) is a biologically heterogeneous malignancy with relatively uniform treatment paradigms. This review aims to assess the growing role of Minimal Residual Disease (MRD) assessment in facilitating response-adapted therapeutic decision making to individualize therapy in MM.

**Recent Findings:**

MRD has been repeatedly demonstrated to provide strong prognostic information, superseding traditional IMWG response criteria. The use of MRD to modulate therapy remains controversial. Here, we review the existing landscape of MRD-adapted trial designs in both induction/consolidation and maintenance settings, including recent data from influential studies and retrospective analyses. We navigate existing data, leverage the increased resolution of longitudinal MRD assessments, and comment on trials in progress to explain our current utilization of MRD in the clinic.

**Summary:**

MRD transcends traditional response assessments by providing a window into disease-treatment interaction over time. As a strong patient-level surrogate, MRD has limited current use in individualizing treatment, but is poised to comprehensively shape treatment strategies at many key points in a patient’s MM course.

## Introduction

Minimal (Measurable) Residual Disease (MRD) in multiple myeloma (MM) refers to the disease burden that remains following therapeutic intervention that may be detectable by highly sensitive assays. It has emerged as one of the strongest prognostic metrics for MM in all disease settings [1–6]. In this modern age of therapy, MRD status has been seen to outperform and possibly invalidate uniform response criteria [1, 7–11]. Though there has been debate as to its role as a surrogate for progression-free survival (PFS; itself a surrogate for overall survival) and its validity as an endpoint for the purposes of regulatory drug approval in clinical trials [12–20], there is little argument that those that achieve MRD-negativity and sustain deep responses have unparalleled long-term outcomes. Past prognostication, however, there is little consensus as to the clinical application of MRD as a tool to adapt therapy.

MRD assays, utilizing flow cytometry to directly count neoplastic cells, or next-generation sequencing (NGS) to quantify remaining clonal immunoglobulin gene sequences, have evolved as the most sensitive assays for detecting and quantifying residual myeloma cells. Considering the variety of assays, laboratory protocols, and technologies, some current assays focused on maximizing input material can achieve sensitivity as deep as 10^–7^ or 1 tumor cell in 10,000,000 cells [12, 21]. The detection of such small quantities of cells can be reflective of underlying disease biology and provide insights into disease-treatment kinetics.

MM is increasingly seen as an extremely heterogeneous disease [22], yet most patients are treated homogenously according to their fitness. Furthermore, diagnostic criteria are such that those with disease-causing end-organ damage are treated alongside those meeting only biochemical criteria for diagnosis. Overall, our current paradigms can be argued to result in overtreatment for some with relatively biologically indolent disease and undertreatment (or ineffective treatment) for some with the most aggressive presentations. Both groups will then generally undergo continuous [maintenance] therapy as a standard of care independent of response to therapy. Measurement of MRD at various landmarks may allow for the modulation of therapeutic approaches to better meet the individualized needs and disease characteristics of each patient. To this end, a growing body of evidence and clinical studies have sought to evaluate the role of MRD in adapting therapy. In this review, we will summarize the current body of clinical studies that have evaluated MRD-adapted therapy, and our current use of MRD to aid in therapeutic decision-making.

## Readily Available and Actionable MRD Assays

A variety of assays designed to measure MRD are currently under investigation and development. Assays include flow cytometry, mass spectrometry, and next-generation sequencing (NGS) and analytes range from individual cells to [circulating] tumor DNA to clonotypic peptides [6]. While most clinically relevant testing is performed on bone marrow specimens, there is great excitement about the evolution of peripheral blood-based (i.e., minimally invasive) diagnostics. Further still, highly sensitive imaging techniques are in development as adjuncts for MRD assessment given that localized marrow-based assays may underrepresent the spatial heterogeneity of MM [6, 23]. Though there is much to be excited about, the strongest evidence and most widely available combination of assessments include either marrow-based flow cytometry or next-generation sequencing [24]. These will be the focus of discussion here as assays with which we may act in the clinic.

Clinically available NGS approaches leverage the unique immunoglobulin gene sequences of each patient’s plasma cell clone. This requires a baseline sample to identify the dominant sequence which will be monitored in the post-treatment samples. Identifying tumor-specific sequences can pose challenges, as baseline sequencing might not identify a suitable clone for monitoring in initial samples, owing to the random occurrence of somatic hypermutation, even in cases with a high disease burden. Nevertheless, incorporating additional primer sets in the assay helps mitigate this issue. This method boasts an impressive sensitivity with an ability to identify one in one million (10^–6^) cells, and, as of now, the Adaptive ClonoSEQ is the only FDA-approved assay. Principally, we favor the use of NGS when available, specifically because it is the only standardized assay that is generalizable across institutions and contextualized to many modern clinical trials [1, 3, 11, 24–26].

Multiparametric Flow Cytometry (MFC) remains a mainstay of MRD assessment. The Euro Flow consortium developed a two-tube, eight-color flow assay to enable the analysis of 10 million cells with a significant limit of detection of 2 × 10^–6^ (LOD). MFC offers several advantages, including wide applicability with in-house performing in several academic centers, feasibility without requiring a baseline diagnostic sample, and a rapid turnaround time of approximately 3 to 4 h, However, it relies on the quality of the sample and demands a high level of expertise[1, 26, 27]. There is significant value in this capability especially when a baseline sample cannot be obtained with which to identify a baseline clonotypic immunoglobulin sequence for NGS. When interpreting an assay, at the least, the surface (and cytoplasmic) markers tested, LOD, and the number of leukocytes counted must be reported. The LOD is dependent on the input and conventional flow-based approaches will require at least 3–10 million cell acquisitions, depending on the approach, to achieve between 6 and 2 cells in 1 million (6 × 10^–6^ to 2 × 10^–6^). Another comparable method is the MSKCC 10-color single-tube that integrates the surface and cytoplasmic marker, thereby reducing additional cost and labor burden. It requires at least 3 million cell acquisitions to attain a sensitivity level of 6 cells in 1 million (6 × 10^–6^) [28].

Finally, both NGS and flow-based approaches generally have good concordance. The Forte clinical trial demonstrated 86% and 78% concordance for MFC and NGS at 10^−5^ and 10^–6^ respectively [29]. For both, if the goal of the assay is for clinical decision-making, it is imperative to obtain the highest quality sample. The “first pull” of an aspirate should be utilized, as subsequent pulls are subsequently more hemodilute and likely to be insufficiently cellular and/or interpreted as falsely negative. Many modern clinical trials with MRD endpoints use a protocol-specified first pull.

PET/CT continues to be a valuable imaging adjunct to the MRD assessment method for evaluating, para-medullary, and single focal lesions that may not be detected in bone marrow aspirate, nonetheless, false negatives can occur in ~ 10% of cases. This can be attributed to low hexokinase enzyme activity, which can reduce the uptake of FDG (fluorodeoxyglucose) in myeloma cells [1, 11]. PET/CT may safeguard against false-negative MRD testing, though with adequate marrow sampling (input) and deeper MRD assessment, the value of PET/CT may be lower. In fact, in recent reports, there has been little discordance between PET and MRD at 10^–5^—10^–6^ sensitivity suggesting there may be a more limited role to combined assessment [30]. While PET-CT generally remains the standard for imaging post-response, whole-body diffusion-weighted MRI (DW-MRI) has strong utility in capturing focal lesions that might have increased relevance in the MRD setting [26]. For example, Rasche et al. [31] compared flow cytometry, FDG-PET/CT, and DWI on 168 patients who achieved a complete response following first-line or salvage treatment. Their study revealed that DW-MRI identified a higher percentage of patients with residual focal lesions compared to FDG-PET/CT (21% vs. 6%, respectively). These remaining focal lesions have been linked to shorter PFS. Although further data is needed, DWI-MRI may yet supplant PET/CT with enhancement in detection of residual disease. Given the limitations of PET-CT in myeloma [32], further sensitive imaging assays are in development. In the future, ImmunoPET imaging with tracers such as ^89^Zr-DFO-daratumumab and 68 Ga-pentixafor may provide better resolution for residual loci of disease [33–35]. For now, in current practice, we obtain concurrent PET/CT with MRD assessment when a therapeutic decision is being considered.

## Depth Required for Decision-Making

Even with the variability in the depth of assays in various reports, MRD at any depth is prognostic. The current IMWG definition of MRD negativity is set at a threshold of 10^–5^. However, survival outcomes have seen the greatest improvement when utilizing a sensitivity of 10^–6^ as opposed to 10^–4^ and 10^–5^ thresholds, reflecting the higher discriminatory effects of the deepest clinical responses [4]. In the final report of the MASTER trial [18], the investigators noted that using an MRD threshold of 10^–6^ had more discriminatory power for PFS. Even venturing to extremely deep levels can reveal residual disease that otherwise would have been missed. Recently, the assessment of CD-138-enriched bone marrow using clonoSEQ to attain a sensitivity of 10^–7^ is currently under prospective evaluation in conjunction with mass spectrometry, employing automated Matrix-assisted laser desorption ionization–time-of-flight mass spectrometry (MALDI-TOF) and liquid chromatography and the analysis of cell-free DNA (cfDNA) in peripheral blood [12]. Achievement of sustained MRD-negativity, across time, can mitigate the uncertainties of depth of assessment and affords increased prognostic resolution [15, 36]. Given the requisite time course for measuring sustained MRD-negativity, we find its best potential in modulating continuous therapy.

## MRD to Individualize Treatment Decisions

Increasingly, we are gaining the resolution to characterize the enormous heterogeneity and individual basis of multiple myeloma [22]. Even among disease classified by established canonical translocations and hyperdiploidy that are otherwise used to stratify risk, on an individual patient level, there is evidence of genomic and immune interplay that influences treatment response and outcomes [22, 37, 38]. While traditional FISH has well-established prognostic relevance at the trial level and in retrospective data, it lacks granularity in estimating each patient’s unique disease-treatment interactions. For example, one may not infrequently observe a patient with no high-risk FISH features who nevertheless experiences progression on induction therapy or conversely the patient on their fifth year of lenalidomide maintenance despite t(4;14) translocated disease. To these ends, MRD can assist the clinician in encapsulating disease-treatment interactions into a single assay relevant to an individual patient. This property is otherwise known as patient-level surrogacy; an established characteristic of MRD assessment in MM [39]. In the following sections, we summarize recent studies for which we base our current practical use of MRD assessment (Table 1) and discuss studies for which we eagerly await data (Table 2).

**Table 1:** Existing data to guide MRD-guided decision-making

Trial	Study overview	MRD usage	Patient number	MRD-adapted therapeutic strategy	Technique and sensitivity of MRD detection	Outcomes
Induction/Consolidation:						
NCT01191060 (IFM-2009) (Randomized Phase III)	VRd plus frontline vs salvage ASCT; NDMM	Long-term follow-up analysis; MRD at start maintenance	700	Usage of frontline ASCT	MFC (10^–4^) NGS (10^–6^)	80% relative risk reduction conferred by MRD-negativity, PFS similar in both treatment groups for MRD-negative patients
NCT02969837 (Derman et al.) (Phase II single-arm)	Elo-KRd without ASCT in NDMM	MRD- at 8 and 12 cycles ➔ Elo-Rd MRD + at 8 and MRD- at 12➔ 6 × Elo-KRd and then Elo-RD MRD + at 8 and at 12➔ 12 × Elo-KRd and then Elo-RD;	46	De-escalation/Length of frontline combination therapy	NGS (10^–6^)	3-year PFS and OS rates of 92% and 100%, respectively, for those who de-escalated therapy after MRD-negativity at C8
NCT03224507 (MASTER) (Phase II single-arm)	Up to 12 cycles of Dara-KRd with ASCT after 4 cycles	MRD at 4 cycles Dara-KRd, post-ASCT, and each of 2 × dara-KRd consolidation. After 2 × consecutive MRD- ➔treatment-free surveillance	123	De-escalation/Length of Frontline Therapy	NGS (10^–5^)	71% reached two consecutive MRD negativity, entering treatment-free surveillance. The two-year progression-free survival was 87%. Less suitable for patients with 2 + HRCA
Maintenance:						
Mohan et al (retrospective cohort)	Post-ASCT (IMiD plus PI) maintenance	Long-term follow-up analysis	568	Duration of Maintenance	MFC (10^−5^) NGS (10^–6^)	At 10-year follow-up, 39% of MRD- patients converted to MRD + , mostly in the first 5 years and 70% of them had a clinical relapse after a median 1 year
Myeloma XI (Phase III)	Multi-stage; post-ASCT lenalidomide vs observation	Long-term follow-up analysis	1,248	Duration of lenalidomide maintenance	MFC (4 × 10^−5^)	At 3-year landmark analysis, lack of statistically significant benefit of continued maintenance in MRD- patients
NCT02538198 (Phase II single arm)	MRD dynamics on continuous lenalidomide; no induction/ASCT criteria	Pre-specified Prospective MRD analysis; Yearly MRD up to 5 years	108	Duration of lenalidomide maintenance	MFC (10^–5^)	No progression events in those who maintained sustained MRD negativity at the 2-year landmark
NCT02181413 / NCT02312258 (TOURMALINE MM3 & MM4) (Phase III)	Ixazomib maintenance vs observation;	Long-term follow-up analysis; MRD assessed at (0,14,24 months)	1280	Importance of MRD dynamics; intervention with MRD resurgence?	MFC (4 × 10^−6^)	MRD- at enrollment had similar PFS with or without ixazomib. Half of MRD resurgence in first 2 years; associated with worse outcome
GEM2014MAIN (Phase III)	IRd vs RD post-ASCT in NDMM; MRD-negative at 2 years can cease therapy	Maintenance cessation	332	Duration of maintenance	MFC	MRD-negativity at 2 years with therapy cessation had a better outcome than MRD + and an additional 3 years of extended len. Data is not mature

**Table 2:** Future clinical trials

Trial	Design	MRD Usage	Target patient enrolment	MRD-Adapted Question Addressed	Technique and sensitivity of MRD detection
Induction/Consolidation:					
MASTER-2 trial (NCT05231629)	Dara-VRd induction; randomization based on MRD- to Dara-VRd/Dara-R or ASCT/Dara-R; MRD + to ASCT/Dara-Tec or ASCT/Dara-R	Response-Adapted Consolidation	300	Escalation or De-Escalation of Consolidation; Can alternative consolidation/class-switch (BCMA-T-cell-Redirection) improve MRD + outcomes?	NGS (10^–5^)
NCT04934475 (MIDAS)	Isa-KRd; randomization based on MRD- to Isa-KRd/R or ASCT/Isa-KRd/R; MRD + to ASCT/Isa-KRd/Isa-Iberdomide vs. ASCTx2/Isa-Iber	Response-Adapted Consolidation	716	Escalation or De-Escalation of Consolidation; Can alternative CELMOD maintenance overcome resistance to IMiD induction (i.e., residual disease)?	NGS (10–^5^)
Maintenance:					
NCT03901963 (AURIGA)	Post ASCT maintenance with Dara-R vs R in MRD +	MRD-adapted maintenance assignment	214	Can combination maintenance eliminate residual disease post-frontline?	NGS (10^–5^)
GEM-TECTAL (HR NDMM)	Dara-VRdx4/Tec-Darax6; MRD- to Tec-Dara and MRD + to Tal-Dara	MRD-adapted maintenance assignment	30	Can alternative T-cell Redirection (GPRC5D) maintenance overcome resistance to BCMA T-cell redirection induction (i.e., residual disease)?	MFC (10^–6^)
NCT03710603 (PERSEUS)	VRd ± dara/ASCT and Dara-R or R maintenance; MRD- with 2 years of Dara-R maintenance stop Dara	Maintenance De-escalation	709	Combination Maintenance De-escalation	NGS (10^–5^)
NCT02659293 (ATLAS)	Post-ASCT maintenance with KRD vs R; every 6 months MRD- continue with R in KRD arm	Maintenance De-escalation	180	Combination Maintenance De-escalation	NGS (10^–5^)
NCT04071457 (DRAMMATIC/ SWOG1803)	Post-ASCT maintenance with Dara-R vs R; After 2 years MRD + continue therapy, MRD- randomized to continuation vs cessation	Maintenance Cessation	1100	Cessation of maintenance	NGS (10^–5^)
NCT04221178	MRD- × 3 years can enroll and cease maintenance	Maintenance Cessation	50	Can maintenance be stopped with sustained MRD-negativity	MFC/NGS (10^–5^)
NCT04108624 (MRD2STOP)	1 year of maintenance and MRD- cease maintenance	Maintenance Cessation	56	Can maintenance be stopped with sustained MRD-negativity	NGS (10^–6^)
ISCRTN4684186 (RADAR)	RCyBorD × 4/ASCT and A) MRD- = Isa × 12 and cessation vs continuation; B) Std risk & MRD + = R vs VRd/R vs Isa-R vs Isa-VRd/Isa-R	Maintenance Cessation/ Maintenance Escalation	1400	Cessation of maintenance with sustained MRD-negativity; Escalation of therapy for residual disease	MFC (10^–5^)
NCT04513639 (REMNANT)	VRdx4/ASCT/VRdx4; MRD- randomized to Dara-Kd early (at MRD resurgence) or late (at relapse)	Early Intervention	391	Does early intervention at MRD resurgence improve outcome?	MFC (10^–5^)

Clinical trials were chosen through a search on http://clinicaltrials.gov, as of February 2024

## MRD-Guided Treatment Modulation

### The Induction/Consolidation Setting

While many studies have used MRD as a primary endpoint, few have incorporated MRD as part of the study design to investigate treatment efficacy, duration, and/or the transition to maintenance therapy.

One of the most influential recent studies, the MASTER trial (NCT03224507) [18] was a multicenter single-arm phase II study in the US. Here, a response-adapted platform was utilized to modulate frontline therapy. In 123 NDMM patients treated with Daratumumab, Carfilzomib, Lenalidomide, and Dexamethasone (Dara-KRD), MRD was tested post-induction (4 cycles), post autologous hematopoietic stem-cell transplantation (post-ASCT) and every 4 cycles of consolidation (maximum 8 cycles). Participants who reached 2 consecutive MRD-negative tests stopped treatment and began observation with MRD surveillance (MRD-SURE). 71% reached 2 consecutive MRD negativity, entering treatment-free surveillance. The two-year progression-free survival was 87%. Among those who achieved MRD-SURE, the 24-month cumulative incidence of progression after stopping therapy was 9% for individuals with no high-risk chromosomal abnormalities (HRCAs) such as t(4;14), t(14;16), or del(17p), 9% for those with one high-risk (HRCA), and notably, 47% for those with two or more HRCAs. Notably, there was no strong association between achieving MRD negativity after induction therapy or post-ASCT and progression-free survival, even with using a threshold of 10^–6^.

With longer follow-up, MASTER also showed that MRD resurgence precedes disease progression, highlighting the necessity to study early intervention. Some issues with the study include that with MRD-SURE, the time interval between negative MRD tests may have been too short to capture MRD dynamics and may have led to premature de-escalation of therapy. This played the largest role for those with the highest risk of disease in the study (2 + HRCA) who were not well-served by cessation. The suggestion here is that at least for standard-risk disease, MRD-adapted induction and consolidation is feasible and efficacious. However, we would also argue that in those with ultra-high risk disease, even lengthening the duration of the same therapies may not be the answer to overcoming intrinsically biologically resistant and aggressive disease features. With this in mind, these patients likely would still have poor outcomes with more extended therapy and, in the future, will likely benefit from more aggressive or multimodal (e.g., immunotherapeutic) combinatorial approaches [40].

A multicenter phase II clinical trial (NCT02969837) [41] conducted by Derman and colleagues in the US enrolled 46 NDMM patients treated with elotuzumab and weekly carfilzomib, lenalidomide, and dexamethasone (Elo-KRD) without ASCT. MRD (10^–6^) by NGS was used to guide the duration of Elo-KRD and the transition to Elo-RD (no carfilzomib) until disease progression. 19 out of 43 (44%) patients achieved two consecutive MRD-negative assay after cycles 8 and 12 and were transitioned to maintenance. Patients who achieved MRD-negativity at 12 cycles received 6 additional Elo-KRD cycles and patients who remained positive after 12 cycles received an additional 12 Elo-KRD cycles before transitioning to maintenance. Patients who achieved MRD negativity (10^–6^) by cycle 8 (C8) displayed remarkable 3-year progression-free survival (PFS) and overall survival (OS) rates, with estimates of 92% and 100%, respectively. For those with standard-risk disease, the 3-year PFS rates were 86%, while those with high-risk disease experienced a 3-year PFS rate of 61%. Similarly, the 3-year OS rates for standard-risk patients were 91%, whereas patients with high-risk disease had a 3-year OS rate of 64%. These data demonstrate the feasibility of response-adapted therapy and recapitulate the data seen in MASTER regarding the standard-risk patient population such de-escalation is best suited to assisting. The question remains as to whether those with high-risk disease and residual disease after a course of combination therapy should be subjected to repeated cycles of the same therapy or explore an alternate approach.

Based on these data, as well as robust prospective and retrospective analyses, our main adaptive use of MRD in the frontline setting is to inform the decision to pursue ASCT as a consolidation strategy. We first draw on the data from IFM-2009 and DETERMINATION [42] to indicate that frontline, as opposed to salvage, transplantation affords no additional survival benefit. A retrospective analysis of patients in the IFM-2009 study [43, 44] showed that for patients who had achieved MRD-negativity (10^–4^ sensitivity) before commencing maintenance, PFS outcomes in the transplant vs delayed-transplant arms were similar. Given the results of the IFM-2009 and DETERMINATION studies, we feel that the decision to pursue consolidative ASCT is a discussion between patient and physician considering treatment goals and patient values. The addition of MRD data allows for a more nuanced discussion that indicates that for a patient who has already achieved MRD-negativity, there is little added benefit to consolidative ASCT, and proceeding to maintenance can be elected. To this end, in the MANHATTAN trial, 24 out of 29 (82.7%) patients who achieved MRD-negativity by the end of Dara-KRD induction chose to forego upfront ASCT [45]. The trial also reported 1-year PFS and OS of 98% and 100% respectively.

There are several ongoing clinical trials to adapt treatment toward MRD status in the induction/consolidation setting (Table 2). The MASTER-2 trial (NCT05231629) uses a response-adapted approach to consolidative therapy and helps to answer whether the addition of a novel immune therapy can alter outcomes for those with residual disease after efficacious combination induction. All eligible patients receive Dara-VRD induction for 6 cycles followed by MRD testing. The MRD-negative cohort is randomized to consolidation with either of 3 cycles of Dara-VRd followed by 13 cycles of Dara-R maintenance or ASCT followed by 13 cycles of Dara-R maintenance. The MRD-positive cohort is randomized to ASCT intensification, 3 cycles of Dara-Teclistamab consolidation, and 13 cycles of Dara-Teclistamab maintenance or ASCT intensification, 3 cycles of Dara-R consolidation, and 13 cycles of Dara-R maintenance. The MIDAS trial (NCT04934475) is a phase 3 clinical trial that aims to enroll 761 patients for induction with Isatuxamab-KRD followed by randomization to 4 arms based on MRD status measured by NGS (10^–6^). Subsequent 3-year maintenance includes Revlimid (arm A, B) or Isa-Iberdomide (arm C, D).

### The Maintenance Setting

In current clinical practice, a paradigm of maintenance therapy until progression has become dominant, at least in the US. The rationale for the benefit of lenalidomide maintenance was established in a time of less efficacious frontline therapy. In fact, three main studies leading to our current practice were predicated on induction with therapies not reflective of current practice patterns [46–48]. Given advances in induction regimens, the benefit of continuous maintenance for all patients may be less clear. In the absence of updated randomized data with modern frontline combination therapies, and with the advent of more tolerable and less frequently administered therapies (i.e., antibody-based therapies), MRD-adapted maintenance therapy may better individualize maintenance strategies. MRD is being used as a tool to determine the intensity of maintenance as well as provide insights into de-escalation or even cessation. While we do not yet have strong prospective data to determine whether MRD can be used to adapt the strength or duration of maintenance, it is important to consider the existing evidence for using MRD and MRD dynamics to guide our approach to maintenance de-escalation, especially given the financial and medical toxicities of indefinite maintenance [49–53].

Myeloma XI [16] is a multicenter phase 3 trial in the UK that showed that MRD is a predictor of survival outcomes at 3 and 9 months post-ASCT (ASCT + 3 and ASCT + 9, respectively). 1,248 post-ASCT patients were randomly assigned to lenalidomide maintenance or observation at ASCT + 3. MRD was assessed by flow cytometry (median sensitivity 4 × 10^−5^) before maintenance at ASCT + 3 and ASCT + 9. At ASCT + 3, those who achieved MRD negativity had longer PFS compared to those who did not (44 vs 24 months). Furthermore, those who had MRD negativity at ASCT + 9 had prolonged PFS when compared to those who were MRD positive (50 vs 13 months). OS at 3 years increased from 69.5% of MRD-positive patients to 86.9% of MRD-negative patients. Patients who had a deepening response from MRD positive to MRD negative at ASCT + 9 had similar PFS outcomes as patients who were negative at both points. Valuable information has been gained from a recent analysis [54] with an updated follow-up: Landmark analyses revealed a consistent PFS advantage for all-comers at various time points. However, PFS benefits were no longer statistically significant for those patients with MRD-negativity at 3 years. These data suggest that the magnitude of the benefit of extended maintenance for those patients with the deepest long-term responses may not offset medical and financial toxicities.

In a similar vein, NCT02538198 [15] was a single-arm phase 2 clinical trial in the USA that studied the dynamics of MRD in patients on continuous lenalidomide maintenance. 108 patients underwent annual MRD tests by flow cytometry for up to 5 years (1 × 10^–5^). Most MRD-negative-to-positive resurgences happened within the first 2 years and no progression events were recorded for those who maintained sustained MRD negativity at a 2-year landmark analysis. Additionally, patients who experienced an MRD resurgence exhibited inferior outcomes compared to those who maintained a stable MRD-positive response, signifying imminent disease progression. Importantly, other studies on MRD dynamics below have conversely reported similarly poor outcomes for those with both persistent MRD-positivity and conversion from -negative to -positive state. In a separate study by Mohan et al., [55] a retrospective cohort of 568 patients who were in deep remission [achieved sustained MRD-negativity post-ASCT at least 3 months apart, had negative PET-CT or whole-body MRI and sustained a very good partial response of higher] and were receiving immunomodulator plus proteasome inhibitor for maintenance (IMiD plus PI). The findings revealed that, during a median follow-up of 9.9 years from diagnosis, 61% of patients maintained MRD negativity, while 39% had MRD resurgence at a median of 6.3 years subsequently leading to a clinical relapse within a median of 1.0 years. The study identified that the highest risk of MRD resurgence occurred within the initial 5 years post-treatment and extended up to 15 years from the time of diagnosis. Notably, only 27% of those with MRD resurgence had not experienced clinical relapse at a median follow-up of 9.3 years. These studies highlight both the potential of MRD assessment to detect imminent progression (~ 1 year at 10^–5^) and also call into question the utility of continuous maintenance for those with durable and prolonged sustained MRD-negative responses.

Two simultaneously published analyses examined MRD resurgence [56–58]. In an analysis from the FORTE study, some important findings included that of 118 patients who lost MRD-negativity: 1) 1-year sustained MRD-negativity had been previously achieved by 36%; 2) 16 patients with a recent MRD-negative assay had skeletal/extramedullary relapse without biochemical progression; 3) median time from MRD resurgence to biochemical progression (at 10^–5^) was 22.3 months. Features associated with unsustained MRD-negativity included HRCA, baseline high circulating tumor cell burden, late timing (i.e., post-consolidation) of MRD-negativity, and monotherapy (R) maintenance (vs. KR). The second report was a combined analysis of GEM2012MENOS65 and GEM2014MAIN. GEM2014MAIN^51^ de-escalated maintenance therapy based on MRD status at 2 years. Patients were randomized following GEM2012menos65 (VRD induction with busalfan/melphalan vs melphalan ASCT) onto maintenance with either of Ixazomib Plus Lenalidomide/Dexamethasone (IRd) vs Rd maintenance. At 2 years, those with MRD-negativity by MFC (sensitivity 3 × 10^–6^) would stop maintenance and those with residual disease would continue Rd. Parallel findings to the aforementioned FORTE analysis included: 1) Median PFS from MRD resurgence to progression or death was 39 months; 2) 47% who had MRD resurgence had previously sustained MRD-negativity; and 3) Late achievement of MRD- and 4) high baseline circulating tumor cells were similarly seen to be associated with MRD resurgence. Furthermore, a higher level of MRD at the time of resurgence (i.e.; < 10^–5^, in between ≥ 10^–5^ and < 10^–3^, and ≥ 10^–3^) was associated with worse PFS. Contrary to the FORTE analysis, however, high ISS was associated with MRD resurgence while HRCA (aside from 1q +) was not. Additionally, an interim report of 332 patients on GEME2014MAIN revealed that while there was no PFS benefit to the addition of ixazomib in the maintenance setting, those with MRD-negativity at 2 years that had discontinued therapy had lower rates of relapse than those with positive MRD, despite the fact that the latter group received an additional 3 years of extended therapy [59]. Altogether, these studies emphasize that the duration and the depth of sustained MRD-negativity will need to be revisited depending on baseline risk factors and the nature of any planned intervention (i.e., de-escalation/cessation).

A recent combined analysis of TOURMALINE-MM3 and -MM4 [13], in which transplant-eligible and ineligible patients (total n = 1280), were randomized to 2 years of ixazomib maintenance vs placebo provides more granularity into MRD dynamics on maintenance. Patients with CR (and/or VGPR in MM3) had MRD testing by flow cytometry (estimated sensitivity 4 × 10^−6^ with 5 million cell acquisitions) at randomization, 14 months, and end of treatment (∼24 months). The pooled analysis revealed several important findings. First, patients who were MRD-negative at enrollment had similar outcomes whether they received maintenance or placebo with 2-year PFS rates of 67.2% and 61.7% (p = 0.288). Second, about half of MRD resurgence happened within 2 years of starting maintenance, and resurgence was associated with an increased risk of progression in a 14-month landmark analysis compared to those with sustained MRD-negativity (34.2 vs 75%). Third, those with persistent MRD-positivity at 14 months had the worst outcomes with a PFS rate of 27.6%. This data pertains to upcoming trials investigating early intervention with treatment intensification when minimal residual disease (MRD) reappears. While ixazomib maintenance is no longer a relevant treatment modality, the combined analysis further emphasizes the importance of MRD dynamics, rather than MRD status assessed at a single time point, in the maintenance setting.

An important interim analysis comes from the ATLAS trial [60] in which NDMM patients were randomized following ASCT to maintenance with either up to 36 cycles of KRD or Revlimid alone. The risk and response-adapted study design allowed patients in the KRD arm to de-escalate to lenalidomide monotherapy after cycle 6 provided they had standard-risk cytogenetics and MRD-negativity was reached (IMWG 10^–5^). In the interim analysis of 180 patients, 35 patients met de-escalation criteria and compared to 20 patients in the lenalidomide monotherapy arm, there was a PFS benefit to combination maintenance/de-escalation (HR 0.25) providing evidence that MRD-adapted therapy can be efficacious while reducing excess toxicity of combination regimens.

Looking forward with these de-escalation data in mind, two prospective trials aim to determine the safety and efficacy of maintenance cessation. MRD2STOP [12] allows patients who have received at least 1 year of maintenance and are negative for residual disease by PET-CT, flow (10^–5^), and NGS (10^–6^) to discontinue maintenance under careful observation. Of note, one innovation is the prospective assessment of CD138 enriched bone marrow aspirate with NGS to achieve MRD sensitivity of 10^–7^. Preliminarily, 84% of enrolled patients sustained MRD-negativity at 10^–6^ 1 year after enrollment and discontinuation. In preliminary data from a study being conducted by Korde et al., [14] patients who sustained MRD negativity by MFC for 3 years transitioned to close surveillance with BM MRD testing every 6 months and an annual PET-CT. The rates of sustained MRD negativity at 6 months and 12 months are reported as 94% and 88%, respectively. Here, the protocol recommends that for patients who convert to MRD positivity, [lenalidomide] maintenance should be re-initiated.

Based on the data presented here, we may develop individualized plans for the duration of therapy. Generally, our strategy in both fit and frail patients is to provide continuous [maintenance] therapy until progression. For those patients who feel their quality of life would be improved by ceasing therapy, we use MRD to guide cessation. The strongest evidence as indicated by the above studies, is for modulating the length of lenalidomide maintenance in fit patients. Our first recommendation for those seeking a treatment holiday is to do so on a clinical trial. In the absence of trial availability, for patients with sustained MRD-negativity for at least 2 years (and imaging is negative for active disease) we may stop lenalidomide maintenance under careful observation. Monitoring plans are individualized but at the least consist of serum paraprotein surveillance to monitor for biochemical relapse but ideally include serial marrow MRD assessment.

Further ongoing clinical trials are in place to adapt treatment towards MRD status [17, 55] (Table 2).

## Conclusions

**Fig. 1:**
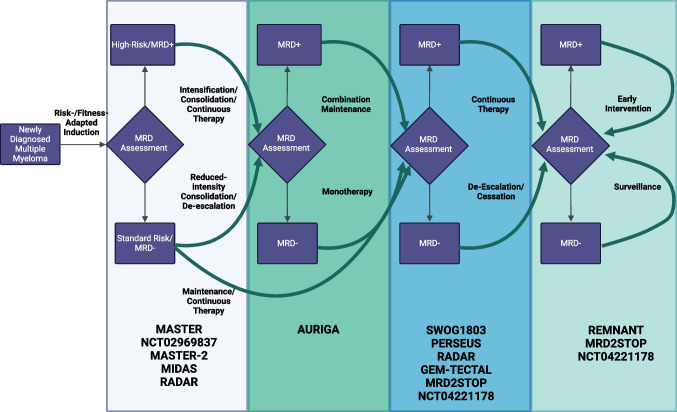
Opportunities and ongoing studies for MRD-adapted therapy

The use of MRD to guide clinical decisions remains a controversial topic, but the current available data and the direction of the field as indicated by studies in progress point towards a future of response-adapted therapy in multiple myeloma. Our current treatment paradigms encourage the relatively uniform treatment of a very heterogeneous disease with a net effect of overtreatment of those with more biologically indolent and treatment-responsive disease states. Conversely, those with aggressive and treatment-resistant disease may be best served by alternative consolidation and maintenance strategies of measurable residual disease. As seen here, the strongest evidence exists for the response-adapted duration of maintenance. With upcoming studies, we may see the adoption of MRD into multiple facets of MM treatment including in de-escalation or cessation of maintenance, omission, or deferral of consolidation (i.e., ASCT), intensification or alternative consolidation for residual disease, and in early intervention for those with early biochemical relapse (i.e., MRD resurgence; Fig. 1). As a patient-level surrogate that encapsulates longitudinal disease-treatment interaction at highly sensitive levels, we look forward to MRD facilitating our move toward individualized treatment.

## Data Availability

No datasets were generated or analyzed during the current study.

## References

[1] Bertamini L, D’Agostino M, Gay F (2021). MRD assessment in multiple myeloma: progress and challenges. Curr Hematol Malig Rep.

[2] Flores-Montero J (2017). Next generation flow for highly sensitive and standardized detection of minimal residual disease in multiple myeloma. Leukemia.

[3] Diamond BT (2021). Defining the undetectable: the current landscape of minimal residual disease assessment in multiple myeloma and goals for future clarity. Blood Rev.

[4] Munshi NC (2020). A large meta-analysis establishes the role of MRD negativity in long-term survival outcomes in patients with multiple myeloma. Blood Adv.

[5] Rajkumar SV (2014). International Myeloma Working Group updated criteria for the diagnosis of multiple myeloma. Lancet Oncol.

[6] Kostopoulos IV, Ntanasis-Stathopoulos I, Gavriatopoulou M, Tsitsilonis OE, Terpos E. Minimal residual disease in multiple myeloma: current landscape and future applications with immunotherapeutic approaches. Front Oncol. 2020;10. Frontiers Media S.A. 10.3389/fonc.2020.00860. 10.3389/fonc.2020.00860 PMC7267070 32537439

[7] Paiva B, San-Miguel J, Avet-Loiseau H (2022). MRD in multiple myeloma: does CR really matter?. Blood.

[8] Cavo M (2022). Prognostic value of minimal residual disease negativity in myeloma: combined analysis of POLLUX, CASTOR, ALCYONE, and MAIA. Blood.

[9] Jiménez-Ubieto A (2021). Validation of the International Myeloma Working Group standard response criteria in the PETHEMA/GEM2012MENOS65 study: are these times of change?. Blood.

[10] Mailankody S (2015). Minimal residual disease in multiple myeloma: bringing the bench to the bedside. Nat Rev Clin Oncol.

[11] Puig N, Cedena M-T, Mateos M-V, Lahuerta JJ, Paiva B, San-Miguel JF, Burgos L. Measurable residual disease in multiple myeloma: ready for clinical practice? J Hematol Oncol. 2020. Accessed: Oct. 09, 2023. [Online]. Available: https://www.ncbi.nlm.nih.gov/pmc/articles/PMC7310444/. 10.1186/s13045-020-00911-4 PMC7310444 32571377

[12] Derman BA (2022). Prospective trial using multimodal measurable residual disease negativity to guide discontinuation of maintenance therapy in multiple myeloma (MRD2STOP). Blood.

[13] Paiva B (2023). MRD dynamics during maintenance for improved prognostication of 1280 patients with myeloma in the TOURMALINE-MM3 and -MM4 trials. Blood.

[14] Korde N (2022). Maintenance therapy cessation for sustained MRD negative multiple myeloma patients. Blood.

[15] Diamond B (2021). Dynamics of minimal residual disease in patients with multiple myeloma on continuous lenalidomide maintenance: a single-arm, single-centre, phase 2 trial. Lancet Haematol.

[16] de Tute RM (2022). Minimal residual disease after autologous stem-cell transplant for patients with myeloma: prognostic significance and the impact of lenalidomide maintenance and molecular risk. J Clin Oncol.

[17] Yong K (2022). Risk-adapted therapy directed according to response (RADAR, UK-MRA myeloma XV) - comparing MRD-guided treatment escalation and de-escalation strategies in patients with newly diagnosed myeloma suitable for stem cell transplantation. Blood.

[18] Costa LJ (2023). Minimal residual disease response-adapted therapy in newly diagnosed multiple myeloma (MASTER): final report of the multicentre, single-arm, phase 2 trial. Lancet Haematol.

[19] Sonneveld P (2019). Bortezomib, lenalidomide, and dexamethasone (VRd) ± daratumumab (DARA) in patients (pts) with transplant-eligible (TE) newly diagnosed multiple myeloma (NDMM): A multicenter, randomized, phase III study (PERSEUS). J Clin Oncol.

[20] Royle K-L (2022). Risk and response adapted therapy following autologous stem cell transplant in patients with newly diagnosed multiple myeloma (RADAR (UK-MRA myeloma XV trial): study protocol for a phase II/III randomised controlled trial. BMJ Open.

[21] Costa LJ (2021). International harmonization in performing and reporting minimal residual disease assessment in multiple myeloma trials. Leukemia.

[22] Maura F et al. Genomic classification and individualized prognosis in multiple myeloma. J Clin Oncol. 2024;JCO.23.01277. 10.1200/JCO.23.01277. 10.1200/JCO.23.01277 PMC11095887 38194610

[23] John L (2023). Resolving the spatial architecture of myeloma and its microenvironment at the single-cell level. Nat Commun.

[24] Kumar S (2016). International Myeloma Working Group consensus criteria for response and minimal residual disease assessment in multiple myeloma. Lancet Oncol.

[25] Derman BA (2023). MRD-guided treatment cessation in multiple myeloma. Lancet Haematol.

[26] Maclachlan KH (2021). Minimal residual disease in multiple myeloma: defining the role of next generation sequencing and flow cytometry in routine diagnostic use. Pathology.

[27] Puig N, Flores-Montero J, Jimenez C, Sarasquete M-E, Garcia-Alvarez M, Prieto-Conde I, Chillon C, Alcoceba M, Gutierrez NC, Oriol A, Medina A, Bladè J, Gironella M, Hernandez MT, Gonzalez-Calle V, Cedena M-T, Paiva B, San-Miguel JF, Lahuerta J-J, Mateos M-V, Rosinol L, Orfao A, Gonzalez M, Garcia-Sanz R, Martinez-Lopez J. Comparison of next-generation sequencing (NGS) and next-generation flow (NGF) for minimal residual disease (MRD) assessment in multiple myeloma. Blood Cancer J. 2020;10(10):108. Accessed: Oct. 03, 2023. [Online]. Available: https://www.ncbi.nlm.nih.gov/pmc/articles/PMC7603393/. 10.1038/s41408-020-00377-0 PMC7603393 33127891

[28] Roshal M (2017). MRD detection in multiple myeloma: comparison between MSKCC 10-color single-tube and EuroFlow 8-color 2-tube methods. Blood Adv.

[29] Oliva S (2020). Multiparameter flow cytometry (MFC) and next generation sequencing (NGS) for minimal residual disease (MRD) evaluation: results of the FORTE trial in newly diagnosed multiple myeloma (MM). J Clin Oncol.

[30] Fonseca R (2023). Integrated analysis of next generation sequencing minimal residual disease (MRD) and PET scan in transplant eligible myeloma patients. Blood Cancer J.

[31] Rasche L (2019). Combination of flow cytometry and functional imaging for monitoring of residual disease in myeloma. Leukemia.

[32] Rasche L (2017). Low expression of hexokinase-2 is associated with false-negative FDG–positron emission tomography in multiple myeloma. Blood.

[33] Ulaner GA, Landgren CO (2020). Current and potential applications of positron emission tomography for multiple myeloma and plasma cell disorders. Best Pract Res Clin Haematol.

[34] Ulaner GA (2020). CD38-targeted immuno-PET of multiple myeloma: From xenograft models to first-in-human imaging. Radiology.

[35] Kraus S (2022). Reduced splenic uptake on 68Ga-Pentixafor-PET/CT imaging in multiple myeloma - a potential imaging biomarker for disease prognosis. Theranostics.

[36] San-Miguel J (2022). Sustained minimal residual disease negativity in newly diagnosed multiple myeloma and the impact of daratumumab in MAIA and ALCYONE. Blood.

[37] Coffey DG (2023). Immunophenotypic correlates of sustained MRD negativity in patients with multiple myeloma. Nat Commun.

[38] Maura F (2023). Genomic and immune signatures predict clinical outcome in newly diagnosed multiple myeloma treated with immunotherapy regimens. Nat Cancer.

[39] Derman BA, Fonseca R (2024). Measurable residual disease and decision-making in multiple myeloma. Hematol Oncol Clin North Am.

[40] Brown S (2021). MUKnine OPTIMUM protocol: a screening study to identify high-risk patients with multiple myeloma suitable for novel treatment approaches combined with a phase II study evaluating optimised combination of biological therapy in newly diagnosed high-risk multiple myeloma and plasma cell leukaemia. BMJ Open.

[41] Derman BA, Kansagra A, Zonder J, Stefka AT, Grinblatt DL, Anderson Jr LD et al. Elotuzumab and weekly carfilzomib, lenalidomide, and dexamethasone in patients with newly diagnosed multiple myeloma without transplant intent. JAMA Oncol. 2022;8(9):1278–1286. Accessed: Oct. 08, 2023. [Online]. Available: https://www.ncbi.nlm.nih.gov/pmc/articles/PMC9305600/. 10.1001/jamaoncol.2022.2424 PMC9305600 35862034

[42] Richardson PG (2022). Triplet therapy, transplantation, and maintenance until progression in myeloma. N Engl J Med.

[43] Perrot A (2018). Minimal residual disease negativity using deep sequencing is a major prognostic factor in multiple myeloma. Blood.

[44] Perrot A (2020). Early versus late autologous stem cell transplant in newly diagnosed multiple myeloma: long-term follow-up analysis of the IFM 2009 trial. Blood.

[45] Landgren O (2021). Safety and effectiveness of weekly carfilzomib, lenalidomide, dexamethasone, and daratumumab combination therapy for patients with newly diagnosed multiple myeloma: the MANHATTAN nonrandomized clinical trial. JAMA Oncol.

[46] Attal M (2012). Lenalidomide maintenance after stem-cell transplantation for multiple myeloma. N Engl J Med.

[47] Palumbo A (2012). Continuous lenalidomide treatment for newly diagnosed multiple myeloma. N Engl J Med.

[48] McCarthy PL (2012). Lenalidomide after stem-cell transplantation for multiple myeloma. N Engl J Med.

[49] Jones JR (2023). Maintenance lenalidomide in newly diagnosed transplant eligible and non-eligible myeloma patients; profiling second primary malignancies in 4358 patients treated in the Myeloma XI Trial. EClinicalMedicine.

[50] Marchetti M, Gale RP, Barosi G (2021). Cost-effectiveness of post-autotransplant lenalidomide in persons with multiple myeloma. Mediterr J Hematol Infect Dis.

[51] Diamond B (2023). Tracking the evolution of therapy-related myeloid neoplasms using chemotherapy signatures. Blood.

[52] Geyer MB (2023). Lenalidomide-associated B-cell ALL: clinical and pathologic correlates and sensitivity to lenalidomide withdrawal. Blood Adv.

[53] Nadeem O (2023). Phase II trial of daratumumab, bortezomib, lenalidomide and dexamethasone in high-risk smoldering multiple myeloma. Blood.

[54] Pawlyn C (2022). Defining the optimal duration of lenalidomide maintenance after autologous stem cell transplant - data from the myeloma XI trial. Blood.

[55] Kendrick S, Szabo A, Yarlagadda N, Atwal D, Pandey Y, Roy A, Parikh R, Lopez J, Thanendrarajan S, Schinke C, Alapat D, Mohan M, Tian E, Tricot G, van Rhee F, Zangari M, Sawyer J. Clinical implications of loss of bone marrow minimal residual disease negativity in multiple myeloma. Blood Adv. 2021;6(3):808–817. Accessed: Oct. 04, 2023. [Online]. Available: https://www.ncbi.nlm.nih.gov/pmc/articles/PMC8945288/. 10.1182/bloodadvances.2021005822 PMC8945288 34807986

[56] Kumar S (2024). Don’t let the genie out of the bottle!. Blood.

[57] D’Agostino M (2024). Predictors of unsustained measurable residual disease negativity in patients with multiple myeloma. Blood.

[58] Guerrero C (2024). Predictors of unsustained measurable residual disease negativity in transplant-eligible patients with multiple myeloma. Blood.

[59] Rosinol L (2021). Ixazomib plus lenalidomide/dexamethasone (IRd) versus lenalidomide /dexamethasone (Rd) maintenance after autologous stem cell transplant in patients with newly diagnosed multiple myeloma: results of the Spanish GEM2014MAIN trial. Blood.

[60] Dytfeld D (2023). Carfilzomib, lenalidomide, and dexamethasone or lenalidomide alone as maintenance therapy after autologous stem-cell transplantation in patients with multiple myeloma (ATLAS): interim analysis of a randomised, open-label, phase 3 trial. Lancet Oncol.

